# Phenytoin Inhibits Cell Proliferation through microRNA-196a-5p in Mouse Lip Mesenchymal Cells

**DOI:** 10.3390/ijms22041746

**Published:** 2021-02-09

**Authors:** Hiroki Yoshioka, Sai Shankar Ramakrishnan, Akiko Suzuki, Junichi Iwata

**Affiliations:** 1 Department of Diagnostic & Biomedical Sciences, School of Dentistry, The University of Texas Health Science Center at Houston, Houston, TX 77054, USA; Hiroki.Yoshioka@uth.tmc.edu (H.Y.); Sai.Shankar.Ramakrishnan@uth.tmc.edu (S.S.R.); akikosuz925@gmail.com (A.S.); 2 Center for Craniofacial Research, The University of Texas Health Science Center at Houston, Houston, TX 77054, USA; 3 MD Anderson Cancer Center UTHealth Graduate School of Biomedical Sciences, Houston, TX 77030, USA

**Keywords:** cleft lip, microRNA, craniofacial development, environmental factor, phenytoin, gene regulation

## Abstract

Cleft lip (CL) is one of the most common birth defects. It is caused by either genetic mutations or environmental factors. Recent studies suggest that environmental factors influence the expression of noncoding RNAs [e.g., microRNA (miRNA)], which can regulate the expression of genes crucial for cellular functions. In this study, we examined which miRNAs are associated with CL. Among 10 candidate miRNAs (miR-98-3p, miR-101a-3p, miR-101b-3p, miR-141-3p, miR-144-3p, miR-181a-5p, miR-196a-5p, miR-196b-5p, miR-200a-3p, and miR-710) identified through our bioinformatic analysis of CL-associated genes, overexpression of miR-181a-5p, miR-196a-5p, miR-196b-5p, and miR-710 inhibited cell proliferation through suppression of genes associated with CL in cultured mouse embryonic lip mesenchymal cells (MELM cells) and O9-1 cells, a mouse cranial neural crest cell line. In addition, we found that phenytoin, an inducer of CL, decreased cell proliferation through miR-196a-5p induction. Notably, treatment with a specific inhibitor for miR-196a-5p restored cell proliferation through normalization of expression of CL-associated genes in the cells treated with phenytoin. Taken together, our results suggest that phenytoin induces CL through miR-196a-5p induction, which suppresses the expression of CL-associated genes.

## 1. Introduction

Cleft lip with or without cleft palate (CL/P) is one of the most common congenital malformations, with a prevalence of 1 in 500 to 1 in 2500 live births worldwide. There are syndromic and nonsyndromic forms of CL/P, often caused by gene mutations, teratogenic exposure, and chromosomal modifications [1,2,3,4]. It is currently estimated that approximately 70% of CL/P cases are nonsyndromic. The etiology of CL/P is thus complex, with multifactorial involvement of genetic and environmental factors along with geographic, racial, and ethnic influences. Previous mouse genetic studies showed that mutations in various genes result in cleft lip (CL), cleft palate, and midfacial cleft. An increasing number of studies suggest that the causative factors for birth defects might be grouped according to shared functions (e.g., cell proliferation, differentiation) and pathways (e.g., growth factor signaling pathways). However, it remains unclear how epigenetic factors regulate CL-associated genes.

Upper lip formation comprises growth and fusion of the facial prominences, including the maxillary process (MP) and the medial and lateral nasal processes (MNP and LNP), which begins on day 26 of gestation in humans and embryonic day (E) 9.5 in mice and is completed by day 48 in humans and E12.5 in mice [5]. Any failure in the development of the MP and MNP leads to CL [5,6]. To identify the mechanism of CL, mouse models have been extensively used, since mouse lip formation and their molecular mechanisms are similar to those of humans.

MicroRNAs (miRNAs) comprise approximately 20–22 nucleotide RNAs that play a crucial role in development and disease through the regulation of genes at the post-transcriptional level [7,8,9]. DICER and DROSHA are key enzymes that generate mature miRNAs [10,11]. Mice with a loss of *Dicer* in cranial neural crest cells, the majority of the cells in craniofacial structures, display severe craniofacial deformities, including cleft palate [12,13,14,15]. In addition, mice with a deletion of miR-17-92 cluster exhibit cleft lip and palate and mandibular hypoplasia [16,17]. By contrast, overexpression of miR-17-92 in cultured mouse palatal mesenchymal cells results in increased cell proliferation [18]. Thus, precise control of miRNA expression is crucial for craniofacial development. An increasing number of studies have demonstrated that miRNA expression is altered in CL/P in humans [17,19,20,21,22]. The polymorphisms in *DROSHA*, miR-140, pre-miR-146a, miR-4260, and the 3′-UTR of the target genes are associated with nonsyndromic CL/P in humans [23,24,25,26,27,28]. Recent studies suggest that environmental factors, including maternal conditions (tobacco exposure, alcohol consumption, etc.), can alter miRNA expression during embryogenesis [29,30,31]. Therefore, miRNAs might be a biomarker for diagnosis and potential therapeutic targets for the condition. However, the contribution of each miRNA to CL remains mostly unknown.

Phenytoin, an antiseizure drug, has a teratogenic effect during pregnancy. Maternal exposure to phenytoin causes fetal hydantoin syndrome, characterized by CL/P, microcephaly, facial dysmorphism, heart defects, hypoplastic nails and fingers, and growth or mental retardation [32,33,34]. Phenytoin-treated animal models also show a higher incidence of CL/P, incomplete cleft lip with a Simonart’s band formation, or maxillary hypoplasia [35,36,37]. Previous studies indicated that phenytoin exposure induces CL/P through induction of hypoxia [38,39,40], alterations in gene expression [41,42], suppression of RNA or protein synthesis [43], and inhibition of apoptosis at the medial edge epithelium between the MP and MNP [44]. However, it is still unknown how miRNAs induced by phenytoin contribute to CL/P. In this study, we investigated 10 candidate CL-associated miRNAs identified through our bioinformatic analysis. In addition, we evaluated whether the expression of these miRNAs was induced with phenytoin in cultured cells, and whether suppression of cell proliferation by phenytoin treatment could be rescued with normalization of miRNA expression.

## 2. Results

### 2.1. Overexpression of miR-181a-5p, miR-196a-5p, miR-196b-5p, and miR-710 Inhibits Cell Proliferation in Mouse Embryonic Lip Mesenchymal (MELM) and O9-1 Cells

**Figure 1:**
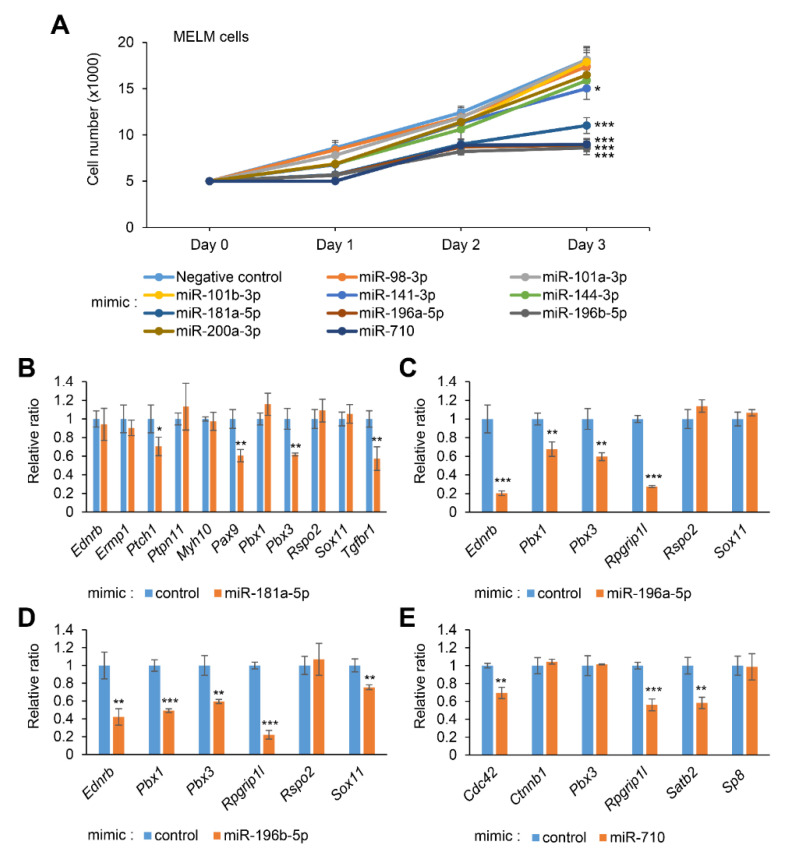
Effect of overexpression of the predicted microRNAs (miRNAs) on proliferation of mouse embryonic lip mesenchymal (MELM) cells. (**A**) Cell proliferation assays with primary MELM cells treated with the indicated miRNA mimic for control, miR-98-3p, miR-101a-3p, miR-101b-3p, miR-141-3p, miR-144-3p, miR-181a-5p, miR-196a-5p, miR-196b-5p, miR-200a-3p, and miR-710. * *p* < 0.05, *** *p* < 0.001. Each treatment group was compared to the control. (**B**–**E**) Quantitative RT-PCR for the indicated genes after treatment of primary MELM cells with control or miR-181a-5p mimic (**B**), miR-196a-5p mimic (**C**), miR-196b-5p mimic (**D**), and miR-710 mimic (**E**). * *p* < 0.05, ** *p* < 0.01, *** *p* < 0.001 versus control (*n* = 6).

To evaluate the role of the candidate miRNAs, primary MELM cells were treated with a miRNA mimic for either miR-98-3p, miR-101a-3p, miR-101b-3p, miR-141-3p, miR-144-3p, miR-181a-5p, miR-196a-5p, miR-196b-5p, miR-200a-3p, or miR-710, which were predicted through our bioinformatic analyses [45]. Among them, miR-181a-5p, miR-196a-5p, miR-196b-5p, and miR-710 mimics significantly inhibited cell proliferation in MELM cells, while miR-98-3p, miR-101a-3p, miR-101b-3p, miR-141-3p, miR-144-3p, and miR-200a-3p mimics failed to inhibit cell proliferation, as shown in Figure 1A. To confirm the effect of each miRNA on cell proliferation, O9-1 cells were also used for the analysis. As Supplementary Figure S1A shows, we found that treatment with either miR-181a-5p, miR-196a-5p, miR-196b-5p, or miR-710 mimic similarly affected the cell proliferation in O9-1 cells. These results indicate that induction of miR-181a-5p, miR-196a-5p, miR-196b-5p, and miR-710 can inhibit cell proliferation in lip mesenchymal cells, leading to CL. 

### 2.2. Overexpression of miR-181a-5p, miR-196a-5p, miR-196b-5p, and miR-710 Downregulates CL-Associated Genes in MELM and O9-1 Cells

To identify CL-associated genes targeted by either miR-181a-5p, miR-196a-5p, miR-196b-5p, or miR-710 mimic, we performed quantitative RT-PCR analysis for the predicted target genes (11 CL-associated genes for miR-181a-5p, 6 CL-associated genes for miR-196a-5p, 6 CL-associated genes for miR-196b-5p, and 6 CL-associated genes for miR-710) in MELM cells after treatment with each miRNA mimic. We found that, among them, expression of four CL-associated genes *(Ptch1*, *Pax9*, *Pbx3*, and *Tgfbr1*) in cells treated with miR-181a-5p mimic, four CL-associated genes (*Ednrb*, *Pbx1*, *Pbx3*, and *Rpgrip1l*) in cells treated with miR-196a-5p mimic, five CL-associated genes (*Ednrb*, *Pbx1*, *Pbx3*, *Rpgrip1l*, and *Sox11*) in cells treated with miR-196b-5p mimic, and three CL-associated genes (*Cdc42*, *Rpgrip1l*, and *Satb2*) in cells treated with miR-710 mimic was significantly downregulated in MELM cells; see Figure 1B‒E. To compare miRNA-gene regulation between MELM and O9-1 cells, we performed quantitative RT-PCR analysis for the predicted target genes in O9-1 cells and found that gene expression was similarly altered in both MELM and O9-1 cells (Supplementary Figure S1B‒E). Thus, most of the target genes of the miRNA were common in MELM and O9-1 cells, while there were some differences. This suggests that subtle differences in cell characteristics may alter the target genes of each miRNA.

### 2.3. Inhibition of miR-181a-5p, miR-196a-5p, miR-196b-5p, and miR-710 Regulates CL-Associated Genes without Affecting Cell Proliferation in MELM and O9-1 Cells

**Figure 2:**
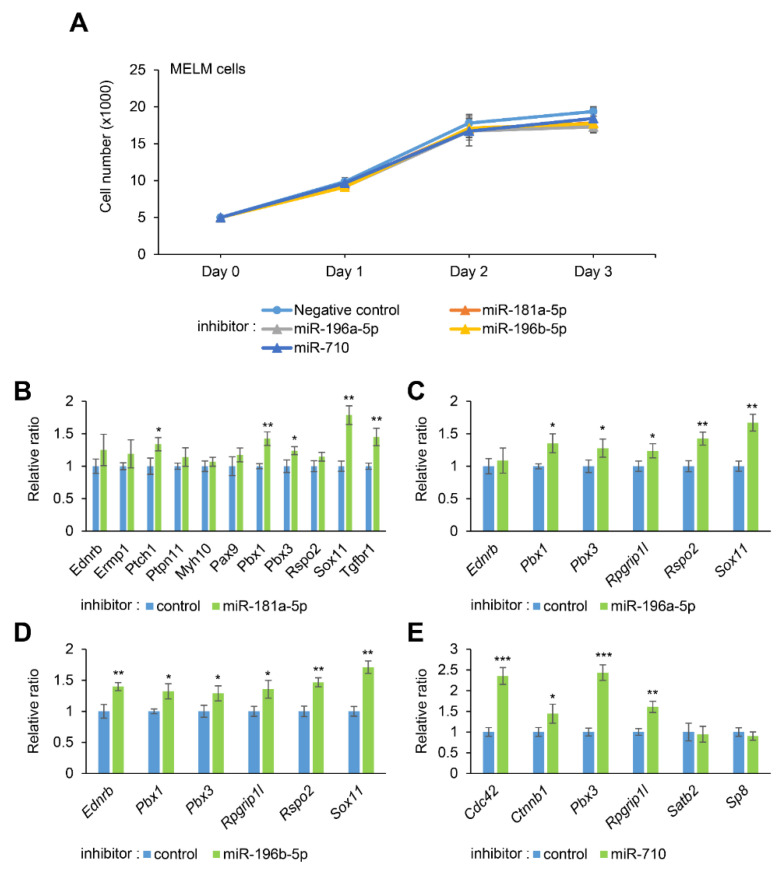
Effect of inhibition of miR-181a-5p, miR-196a-5p, miR-196b-5p, or miR-710 on proliferation of mouse embryonic lip mesenchymal (MELM) cells. (**A**) Cell proliferation assays in primary MELM cells treated with control, miR-181a-5p, miR-196a-5p, miR-196b-5p, or miR-710 inhibitor. (**B**–**E**) Quantitative RT-PCR for the indicated genes after treatment of primary MELM cells with control or miR-181a-5p inhibitor (**B**), miR-196a- inhibitor (**C**), miR-196b-5p inhibitor (**D**), and miR-710 inhibitor (**E**). * *p* < 0.05, ** *p* < 0.01, *** *p* < 0.001 versus control (*n* = 6).

To evaluate the contribution of each miRNA to cell proliferation and gene regulation, we treated cells with a specific inhibitor for either miR-181a-5p, miR-196a-5p, miR-196b-5p, or miR-710. We found that all the inhibitors failed to alter cell proliferation in MELM cells, shown in Figure 2A, and O9-1 cells, shown in Supplementary Figure S2A. By contrast, Figure 2B–E show that the expression of several target genes was upregulated. Expression of five CL-associated genes (*Ptch1*, *Pbx1*, *Pbx3*, *Sox11*, and *Tgfbr1*) in cells treated with the miR-181a-5p inhibitor, five CL-associated genes (*Pbx1*, *Pbx3*, *Rpgrip1l, Rspo2*, and *Sox11*) in cells treated with the miR-196a-5p inhibitor, six CL-associated genes (*Ednrb*, *Pbx1*, *Pbx3*, *Rpgrip1l, Rspo2*, and *Sox11*) in cells treated with the miR-196b-5p inhibitor, and four CL-associated genes (*Cdc42*, *Ctnnb1*, *Pbx3*, and *Rpgrip1l*) in cells treated with miR-710 inhibitor was altered in a dose-dependent manner in MELM cells. Similarly, we found that treatment with each inhibitor induced expression of the target genes in O9-1 cells, as seen in Supplementary Figures S2B‒E and S3. Taken together, miR-181a-5p, miR-196a-5p, miR-196b-5p, and miR-710 can regulate the expression of multiple CL-associated genes in a dose-dependent manner in MELM and O9-1 cells (miR-181a-5p regulates the expression of *Ptch1* and *Tgfbr1*; *miR-196a-5p* of *Pbx1*, *Pbx3*, and *Rpgrip1l*; *miR-196b-5p* of *Ednrb*, *Pbx1*, *Pbx3*, and *Rpgrip1l*; and *miR-710* of *Cdc42* and *Rpgrip1l*). We confirmed that these genes contain binding sites for the predicted miRNA with an in silico analysis, shown in Supplementary Figures S4–S7.

### 2.4. Phenytoin Inhibits Cell Proliferation through miR-196a-5p Induction in MELM and O9-1 Cells

**Figure 3:**
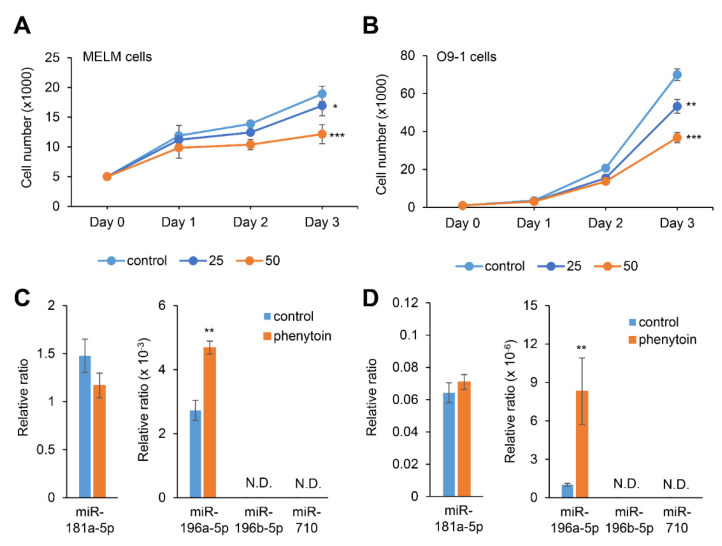
Influence of phenytoin on cell proliferation and microRNA (miRNA) expression in mouse embryonic lip mesenchymal (MELM) and O9-1 cells. (**A**,**B**) Cell proliferation assays with MELM cells (**A**) and O9-1 cells (**B**) treated with 25 or 50 µg/mL phenytoin for 24, 48, and 72 h. * *p* < 0.05, *** *p* < 0.001 versus control (*n* = 6). (**C**,**D**) Quantitative RT-PCR for miR-181a-5p, miR-196a-5p, miR-196b-5p, or miR-710 after treatment of MELM cells (**C**) and O9-1 cells (**D**) with phenytoin for 72 h. ** *p* < 0.01 (*n* = 6). N.D., not detected.

To test whether the expression of candidate miRNAs could be altered with phenytoin, we performed cell proliferation assays in MELM and O9-1 cells treated with 25 or 50 μg/mL phenytoin. We found that cell proliferation was significantly inhibited with phenytoin, in a dose-dependent manner, in these cells, as shown in Figure 3A,B. Interestingly, miR-196a-5p expression was specifically upregulated with phenytoin treatment in both MELM and O9-1 cells, while expression of miR-181a-5p, miR-196b-5p, and miR-710 was not changed or detected under phenytoin treatment, as Figure 3C,D show.

### 2.5. Inhibition of miR-196a-5p Partially Rescues Cell Proliferation in Cells Treated with Phenytoin 

**Figure 4:**
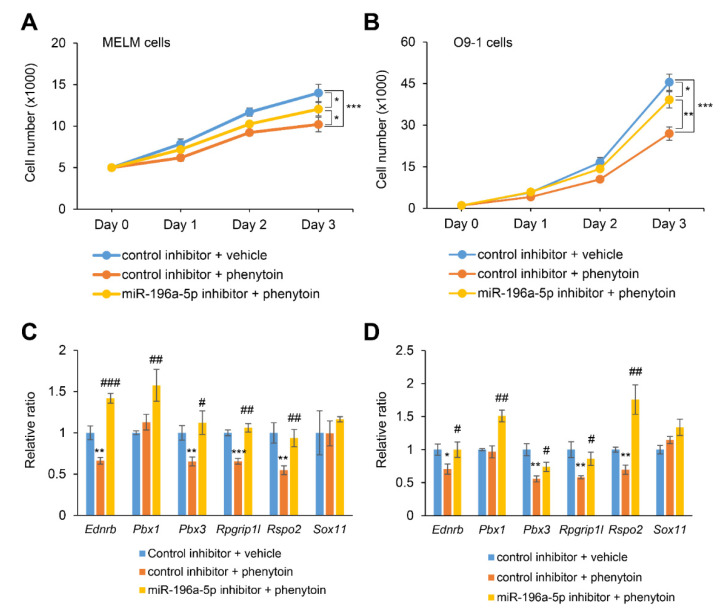
Normalization of miR-196a-5p expression restores phenytoin-induced cell proliferation defects in mouse embryonic lip mesenchymal (MELM) and O9-1 cells. (**A**,**B**) Cell proliferation assays with MELM cells (**A**) or O9-1 cells (**B**) treated with 50 µg/mL phenytoin under miR-196a-5p inhibitor for 24, 48, or 72 h. * *p* < 0.05, ** *p* < 0.01, and *** *p* < 0.001. *n* = 6 per group. (**C**,**D**) Quantitative RT-PCR for the indicated genes after treatment of MELM cells (**C**) or O9-1 cells (**D**) with phenytoin under miR-196a-5p inhibitor for 72 h. * *p* < 0.05, ** *p* < 0.01, and *** *p* < 0.001 versus control inhibitor + vehicle. ^#^ *p* < 0.05, ^##^ *p* < 0.01, and ^###^ *p* < 0.001 versus control inhibitor + phenytoin. *n* = 6 per group.

To evaluate the functional relevance of miR-196a-5p in phenytoin-induced cell proliferation defects, we treated MELM and O9-1 cells with miR-196a-5p inhibitor under phenytoin treatment conditions. Figure 4A,B show that the miR-196a-5p inhibitor partially restored cell proliferation in both MELM and O9-1 cells. In addition, we found that the expression of *Ednrb*, *Pbx1*, *Pbx3*, *Rpgrip1l*, and *Rspo2* was almost fully normalized with miR-196a-5p inhibitor, under phenytoin conditions, in MELM and O9-1 cells, as seen in Figure 4C,D. Taken together, our results indicate that phenytoin inhibits the proliferation of MELM and O9-1 cells through miR-196a-5p expression.

## 3. Discussion

The contribution and distribution of miRNAs and their networks are still mostly unknown in lip formation. To predict functional miRNAs in lip development, we conducted a bioinformatic analysis for CL-associated genes identified through a systematic literature review and a mouse genome informatics (MGI) database search [45]. In the present study, we experimentally evaluated the predicted candidate miRNAs, and found that miR-181a-5p, miR-196a-5p, miR-196b-5p, and miR-710 could suppress the proliferation of MELM cells.

A growing number of miRNA profiling studies shows a significant association of miRNA to nonsyndromic CL/P [20,46]. However, the relationship between CL etiology and miRNA remains unclear. This study found that overexpression of miR-181a-5p, miR-196a-5p, miR-196b-5p, and miR-710 inhibited cell proliferation through suppression of genes associated with mouse CL. The miR-181a-5p, which belongs to the miR-181s family, including miR-181a, -b, -c, and -d, is active in cell proliferation and differentiation in development and cancers. For instance, miR-181 plays a crucial role in the terminal differentiation of skeletal myoblasts by suppressing HOXA11, a transcription factor that induces MyoD [47], and in the differentiation of B-lymphocytes through suppression of Notch signaling [48]. In addition, miR-181a-5p suppresses proliferation and induces apoptosis of glomerular mesangial cells by suppressing *KLF6* and *BCL2* expression via reduction of WNT/β-catenin signaling in human diabetic nephropathy [49] and of glioma cell lines through suppression of the gene encoding for Cyclin B1 [50]. miR-196a-5p and miR-196b-5p, which belong to the miR-196 family of genes and are located on chromosomes 11 and 6, respectively, affect cell proliferation [51]. For instance, miR-196 inhibits the expression of *Hoxb8*, resulting in downregulation of *Shh* expression in mouse forelimb development [52], whereas its overexpression suppresses cell proliferation and migration by suppressing *HOXA9* in human osteosarcoma cell lines [53]. In addition, miR-196a regulates the proliferation of bone marrow mesenchymal stem cells by suppressing *HOXB7* [54]. Although miR-181, miR-196, and miR-710 have not yet been associated with craniofacial developmental defects, our results suggest that dysregulation of these miRNAs may contribute to the pathogenesis of CL. The contributions of these miRNAs will be further evaluated in mice developing CL or in mice overexpressing an individual miRNA.

In the present study, we showed miRNA-mediated gene regulation in MELM and O9-1 cells. We found that miR-181a-5p regulated the expression of *Ptch1* and *Tgfbr1*; miR-196a-5p of *Pbx1*, *Pbx3*, and *Rpgrip1l*; miR-196b-5p of *Ednrb*, *Pbx1*, *Pbx3*, and *Rpgrip1l*; and miR-710 of *Cdc42* and *Rpgrip1l* in a dose-dependent manner. The pre-B-cell leukemia transcription factor (PBX), a member of the TALE/PBX homeobox family, acts as a cofactor of HOX to modulate the DNA binding and transcriptional activity of the HOX transcription factor. The complex composed of PBX::HOX plays essential roles in embryonic development [55,56]. For example, the anterior-posterior segmentation of rhombomeres and pharyngeal arches (PAs) is regulated through the expression pattern of *Hox* genes (namely *Hox* code) [57,58,59]. PBX proteins activate this Hox code. Thus, migration of neural crest cells into the PAs, and consequent craniofacial morphogenesis, depends on the HOX::PBX complex. Therefore, miR-196 may regulate cell proliferation and morphogenesis through downregulation of the HOX::PBX complex. The cell division control protein 42 (CDC42), a Rho small GTPase, plays roles in cell migration, cell cycle regulation, and differentiation [60]. Rpgrip1-like (RPGRIP1L) is located at the transition zone of the primary cilia, acting as a ciliary gatekeeper, and regulates ciliary protein composition [61,62]. Absence of *Rpgrip1l* causes ciliogenesis defects and a failure in the proteolytic process of converting full-length GLI3 to a cleaved repressor form (GLI3R) that disrupts HH signaling [63]. Patched1 (PTCH1), a HH receptor, is located on the primary cilium in the absence of HH ligands; when HH ligands bind to PTCH1, the HH ligand::PTCH1 complex exits from the primary cilium, promoting Smoothened (SMO) to the primary cilium, which results in induction of HH signaling. Thus, these seven genes are all associated with critical signaling pathways related to lip formation, and, therefore, upregulation of miRNAs might induce CL through the dysregulation of these pathways.

We investigated whether and how phenytoin altered miRNA expression that decreased cell proliferation through the expression of genes associated with CL using MELM and O9-1 cells. We found that phenytoin specifically induced miR-196a-5p, which suppressed the expression of genes associated with CL. While expression of some predicted genes, such as *Pbx1* and *Sox11* targeted by miR-196a-5p, was not changed by phenytoin in MELM and O9-1 cells, these genes may be regulated by a combination of other miRNAs or through feedback loops reported in mice and cell lines [64,65,66,67].

A previous study showed that phenytoin treatment suppressed cell proliferation and induced apoptosis in cultured mouse embryonic palatal mesenchymal cells [68]. In addition, treatment of human palatal mesenchymal cells with diphenylhydantoin alters the expression of genes related to the cytoskeleton and cell adhesion [69]. Interestingly, diphenylhydantoin reduces biosynthesis of glycosaminoglycan and collagens in healthy human palatal fibroblasts but not in palatal fibroblasts from individuals with cleft palate [70]. The expression of the special AT-rich sequence-binding protein 2 (*Satb2*), a DNA binding protein associated with transcriptional regulation and craniofacial patterning, is suppressed in embryos exposed to phenytoin [42], and our study shows that there is no dose-dependent regulation of *Satb2* expression by miR-710.

In conclusion, overexpression of miR-181a-5p, miR-196a-5p, miR-196b-5p, and miR-710 inhibits cell proliferation in MELM and O9-1 cells through CL-related genes. Moreover, the phenytoin-induced cell proliferation defect is associated with miR-196a-5p upregulation. Since more than half of the cases of CL/P are nonsyndromic and both environmental and genetic factors are involved in CL/P, the identification of miRNAs influenced by the maternal environment would be clinically relevant to develop a way to prevent and diagnose CL/P. In addition, understanding the miRNA‒gene regulation process will shed light on the mechanism of dynamic change in gene expression during embryogenesis.

## 4. Methods

### 4.1. Animals

C57BL/6J mice were obtained from The Jackson Laboratory. All mice were maintained in the animal facility of UTHealth. The protocol was reviewed and approved by the Animal Welfare Committee (AWC) and the Institutional Animal Care and Use Committee (IACUC) of UTHealth.

### 4.2. Cell Culture

Primary MELM cells were isolated from the anterior half of the maxillary process, a developing lip region, at E11.5, as previously described [45]. The maxillary processes were dissected in sterile Dulbecco’s phosphate-buffered saline (DPBS) without Ca^2+^ and Mg^2+^ and were pooled. To isolate single-cell suspensions, the tissues were incubated with 0.25% trypsin/0.05% ethylenediaminetetraacetic acid (EDTA) for 10 min at 37 °C. MELM cells were cultured in Dulbecco’s Modified Eagle Medium (DMEM) supplemented with 10% fetal bovine serum (FBS), antibiotics (penicillin and streptomycin), L-glutamine, β-mercaptoethanol, and nonessential amino acids. MELM cells were passaged up to two times. Mouse cranial neural crest O9-1 cells (SCC049, Sigma-Aldrich, St. Louis, MO, USA) were cultured under a conditioned medium provided by a mouse embryonic fibroblast cell line called STO cells (CRL-1503, ATCC), as previously described [45].

### 4.3. Cell Proliferation Assay

Cells were plated onto 96-well cell culture plates at a density of 5000 cells (MELM cells) or 1000 cells (O9-1 cells) per well and treated with a mimic for negative control, miR-98-3p, miR-101a-3p, miR-101b-3p, miR-141-3p, miR-144-3p, miR-181a-5p, miR-196a-5p, miR-196b-5p, miR-200a-3p, and miR-710 (mirVana miRNA mimic, Thermo Fisher Scientific, Waltham, MA, USA), or an inhibitor for negative control, miR-181a-5p, miR-196a-5p, miR-196b-5p, and miR-710 (mirVana miRNA inhibitor, Thermo Fisher Scientific), using Lipofectamine RNAiMAX transfection reagent (Thermo Fisher Scientific), according to manufacturer protocol (3 pmol of mimic and 0.3 µL of transfection reagent in 100 µL DMEM per well). Cell proliferation was measured using the Cell Counting Kit 8 (Dojindo Molecular Technologies, Gaithersburg, MD, USA) at 24, 48, or 72 h after each treatment (*n* = 6 per group). 

### 4.4. Quantitative RT-PCR

MELM and O9-1 cells were plated onto a 35-mm dish at a density of 20,000 cells per dish. When the cells reached 80% confluence, the cells were treated with a mimic or inhibitor for the negative control, miR-181a-5p, miR-196a-5p, miR-196b-5p, or miR-710 at 3 pmol in 6 µL of transfection reagent (Lipofectamine RNAiMAX transfection reagent in 2 mL DMEM per dish). One day after treatment, total RNA was extracted with the QIAshredder and miRNeasy Mini Kit (QIAGEN, Hilden, Germany), according to manufacturer instructions. Total RNA (1 µg) from each sample was reverse-transcribed using iScript Reverse Transcription Supermix for qRT-PCR (Bio-Rad, Hercules, CA, USA), and cDNA was amplified with the iTaq Universal SYBR Green Supermix (Bio-Rad) on a CFX96 Touch Real-Time PCR Detection System (Bio-Rad). The amount of each mRNA was normalized by *Gapdh*. The PCR primers used in this study are listed in Supplemental Table S1.

### 4.5. In Silico Analysis for Binding Sites of miR-181a-5p, miR-196a-5p, miR-196b-5p, and miR-710 on the CL-Associated Genes

The mature sequence of miR-181a-5p (5′-AACAUUCAACGCUGUCGGUGAGU-3′), miR-196a-5p (5′-UAGGUAGUUUCAUGUUGUUGGG-3′), miR-196b-5p (5′- UAGGUAGUUUCCUGUUGUUGGG-3′), and miR-710 (5′-UGUGGGGCUGUGGCCAUCAAGAA-3′) and the 3′-UTR of CL-associated genes (*Cdc42*, *Ednrb*, *Pbx1*, *Pbx3*, *Ptch1*, *Rpgrip1l*, and *Tgfbr1*) were analyzed by Clustal Omega to predict a binding site for each miRNA. 

### 4.6. Phenytoin Exposure to Cultured Cells

For cell proliferation assays, the cells were plated onto 96-well cell culture plates at a density of 5000 per well (MELM cells) or 1000 per well (O9-1 cells) and treated with phenytoin (D4505, Sigma-Aldrich) at 25 or 50 μg/mL for 24, 48, or 72 h. For quantitative RT-PCR, the cells were plated onto a 35-mm dish at a density of 25,000 (MELM cells) or 15,000/well (O9-1 cells) and treated with phenytoin (D4505, Sigma-Aldrich) at 50 μg/mL for 72 h.

### 4.7. Rescue Experiment Against Phenytoin Toxicant

MELM and O9-1 cells were plated onto 60-mm dishes at a density of 400,000 per well and 200,000 per well, respectively. After 24 h, the cells were transfected with either miR-196a-5p inhibitor (3 pmol) or control miRNA inhibitor (3 pmol; mirVana, Thermo Fisher Scientific) using Lipofectamine RNAiMAX transfection reagent (Thermo Fisher Scientific), according to manufacturer protocol (12 µL of transfection reagent in 4 mL DMEM per dish). The cells were harvested 24 h after transfection and used for further experiments (cell proliferation assays and quantitative RT-PCR).

### 4.8. Statistical Analysis

A *p*-value less than 0.05 in two-tailed Student’s *t*-tests or post hoc Tukey-Kramer’s test was considered to be statistically significant. For all graphs, data were parametric and represented as mean ± standard deviation (SD).

## Data Availability

All relevant data are within the manuscript and its Supporting Information files. The datasets generated and analyzed during the current study are also available from the corresponding author upon request.

## Supplementary Materials

The following are available online at https://www.mdpi.com/1422-0067/22/4/1746/s1.
