# Supplementary material for: Phenytoin Inhibits Cell Proliferation through microRNA-196a-5p in Mouse Lip Mesenchymal Cells
Source: Int J Mol Sci. 2021 Feb 9;22(4):1746. doi: 10.3390/ijms22041746 (PMC7916186; doi:10.3390/ijms22041746)

Supplementary Information

Supplementary Table S1. Primer pairs used in this study.

Gene	Forward primer	Reverse primer
Cdc42	5'-ATGTGAAAGAAAAGTGGGTGCC-3'	5'-GATGCGTTCATAGCAGCACAC-3'
Ctnnb1	5'-ATGGAGCCGGACAGAAAAGC-3'	5'-CTTGCCACTCAGGGAAGGA-3'
Ednrb	5'-CCCTAAGGGTCTGCATGCTT-3'	5'-GCCACTTCTCGTCTCTGCTTT-3'
Ermp1	5'-GGGCTTCACGAGCTACTATGAC-3'	5'-CTGACATGACCCGGAGAACTTC-3'
Myh10	5'-GCAGAGCTGACATGCTTGAAC-3'	5'-GATGTGTGGTGGCATCTCATG-3'
Pax9	5'-ACCGCTTCTGCACTCTGATG-3'	5'-GGGCAACACAAATGCCTCAT-3'
Pbx1	5'-TGCCACAGAATGAAGCCTGC-3'	5'-CTGAGTTGTCTGAACCTGCACC-3'
Pbx3	5'-CATCGGCGACATCCTCCAC-3'	5'-TGTGAATTCATTACATGCCTGTTCA-3'
Ptch1	5'-TAGCCCTGTGGTTCTTGTCC-3'	5'-TGTGGTCATCCTGATTGCAT-3'
Ptpn11	5'-GGTGAATGACTTCTGGCGGC-3'	5'-CTCTCTGTGTTTCCTTGTCCGA-3'
Rpgrip1l	5'-GACAGATCAGCTTCAGACAGTG-3'	5'-GGCAGGGACATAGGAGTCTC-3'
Rspo2	5'-GGTCTGGAAACCAGAACACGG-3'	5'-GAAGACGCTGTGCTGCTCTTG-3'
Satb2	5'-CCTCAAAATCACACACCAGCA-3'	5'-GGGACCTTGGTGTTGGAACTA-3'
Sox11	5'-CAGCGAGAAGATCCCGTTCA-3'	5'-GGGTCCGTCTTGGGCTTTT-3'
Sp8	5'-TTAAACTTGACTCCGCCGCT-3'	5'-AGCAAAACTAGGCCCGGAAA-3'
Tgfbr1	5'-GGCCGGGCCACAAACA-3'	5'-CTGAAAAAGGTCCTGTAGTTGGG-3'
Gapdh	5'-AACTTTGGCATTGTGGAAGG-3'	5'-ACACATTGGGGGTAGGAACA-3'

Supplementary Figure S1. Effect of overexpression of the predicted miRNAs on proliferation of O9-1 cells. (**A**) Cell proliferation assays with O9-1 cells treated with a miRNA mimic for control, *miR-98-3p, miR-101a-3p, miR-101b-3p, miR-141-3p, miR-144-3p, miR-181a-5p, miR-196a-5p, miR-196b-5p, miR-200a-3p,* or *miR-710*. (**B-E**) Quantitative RT-PCR for the indicated genes after treatment of O9-1 cells with control or miR-181a-5p mimic (B), miR-196a-5p mimic (C), miR-196b-5p mimic (D), and miR-710 mimic (E). *$p<0.05$, **$p<0.01$, ***$p<0.001$ versus control (n=6).

Supplementary Figure S2. Effect of inhibition of miR-181a-5p, miR-196a-5p, miR-196b-5p or miR-710 on proliferation of O9-1 cells. (**A**) Cell proliferation assays with O9-1 cells treated with

the control, miR-181a-5p, miR-196a-5p, miR-196b-5p or miR-710 inhibitor. (**B-E**) Quantitative RT-PCR for the indicated genes after treatment of O9-1 cells with control or miR-181a-5p inhibitor (B), miR-196a- inhibitor (C), miR-196b-5p inhibitor (D), or miR-710 inhibitor (E). *$p<0.05$, **$p<0.01$, ***$p<0.001$ versus control (n=6).

Supplemental Figure 3. Spatiotemporal expression of *miR-181a-5p, miR-196a-5p, miR-196b-5p,* and *miR-710* during lip development. (**A**) Relative expression of *miR-181a-5p, miR-196a-5p,* and *miR-196b-5p* in the maxillary process (MxP) and nasal process (NP) from E9.5 C57BL/6J mice. (**B, C**) Relative expression of *miR-181a-5p, miR-196a-5p, miR-196b-5p,* and *miR-710* in the MxP (B) and NP (C) from C57BL/6J mice from E9.5 to E12.5. *$p<0.05$, **$p<0.01$, ***$p<0.001$ versus control (n=6). N.D., not detected.

Supplementary Figure 4. Putative target site for miR-181a-5p in the *Ptch1* and *Tgfbr1* 3' UTR. (**A, B**) Bioinformatic analysis of the complementarity of miR-181a-5p seed-sequence to the 3' UTR of *Ptch1* (A) and *Tgfbr1* (B).

Supplementary Figure 5. Putative target site for miR-196a-5p in the *Pbx1*, *Pbx3*, and *Rpgrip1l* 3' UTR. (**A-C**) Bioinformatic analysis of the complementarity of miR-196a-5p seed-sequence to the 3' UTR of *Pbx1* (A), *Pbx3* (B), and *Rpgrip1l* (C).

Supplementary Figure 6. Putative target site for miR-196b-5p in the *Ednrb*, *Pbx1*, *Pbx3*, and *Rpgrip1l* 3' UTR. (**A-D**) Bioinformatic analysis of the complementarity of miR-196b-5p seed-sequence to the 3' UTR of *Ednrb* (A), *Pbx1*(B), *Pbx3* (C), and *Rpgrip1l* (D).

Supplementary Figure 7. Putative target site for miR-710 in the *Cdc42* and *Rpgrip1l* 3' UTR. (**A, B**) Bioinformatic analysis of the complementarity of miR-710 seed-sequence to the 3' UTR of *Cdc42* (A) and *Rpgrip1l* (B).

Supplementary Figure 1

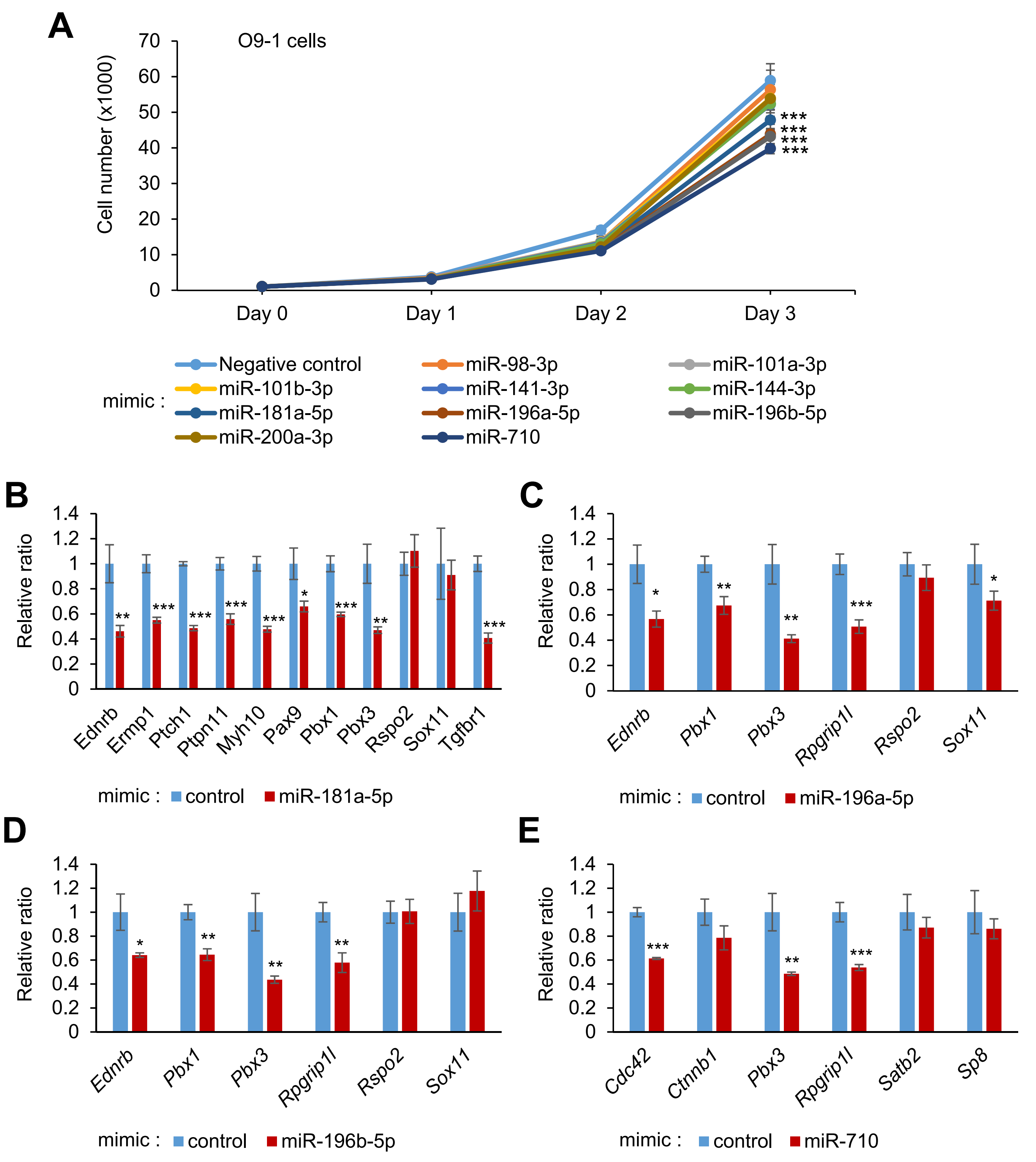

Supplementary Figure 2

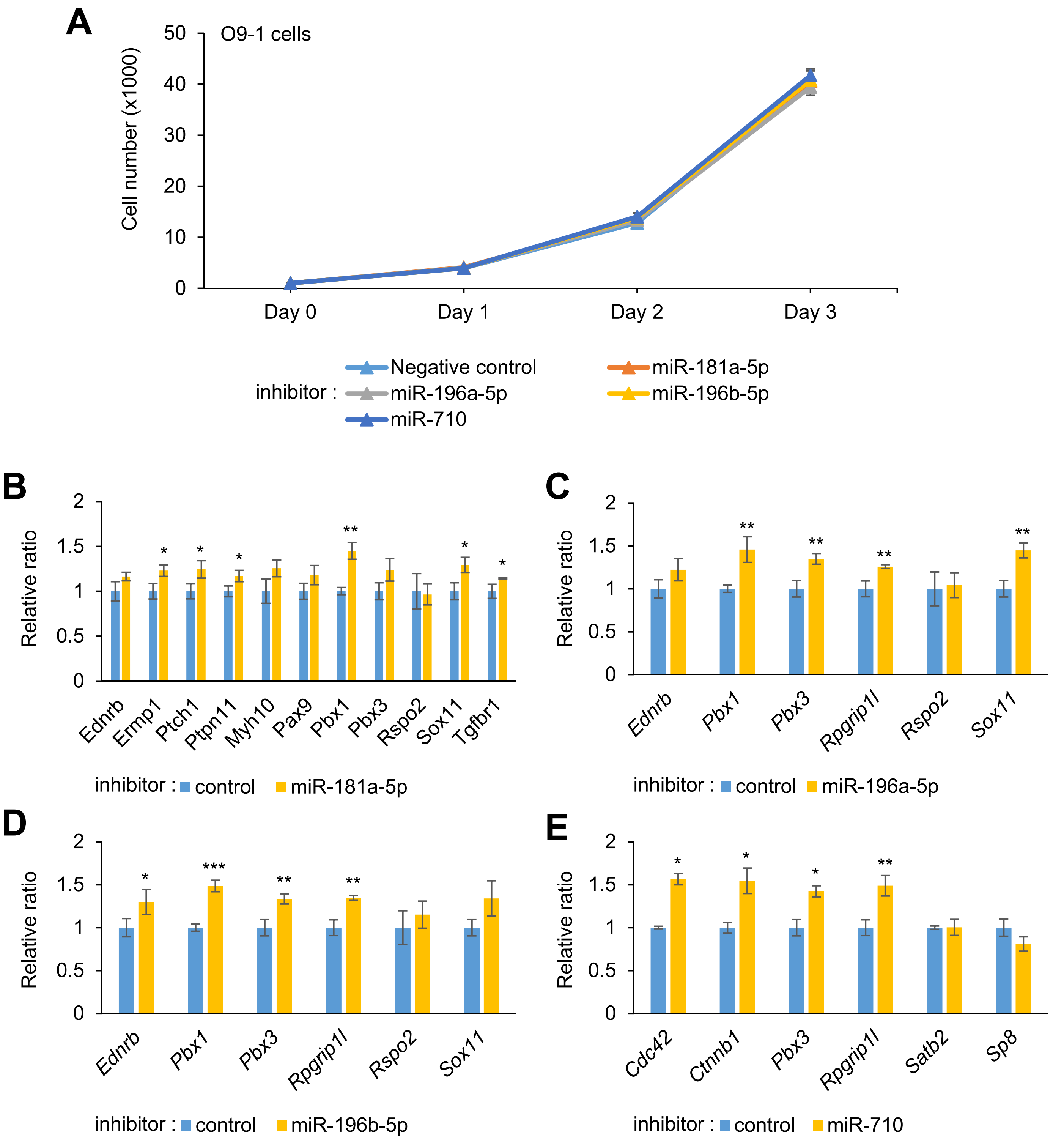

Supplementary Figure 3

A

	E9.5 MxP	E9.5 NP
miR-181a-5p/miR-26a-5p	1.97 ± 0.20	1.91 ± 0.24
miR-196a-5p/miR-26a-5p	6.30 ± 0.11 ($x10^{-3}$)	8.87 ± 1.76 ($x10^{-3}$)
miR-196b-5p/miR-26a-5p	2.44 ± 0.52 ($x10^{-3}$)	7.96 ± 1.37 ($x10^{-4}$)
miR-710/miR-26a-5p	N.D.	N.D.

B

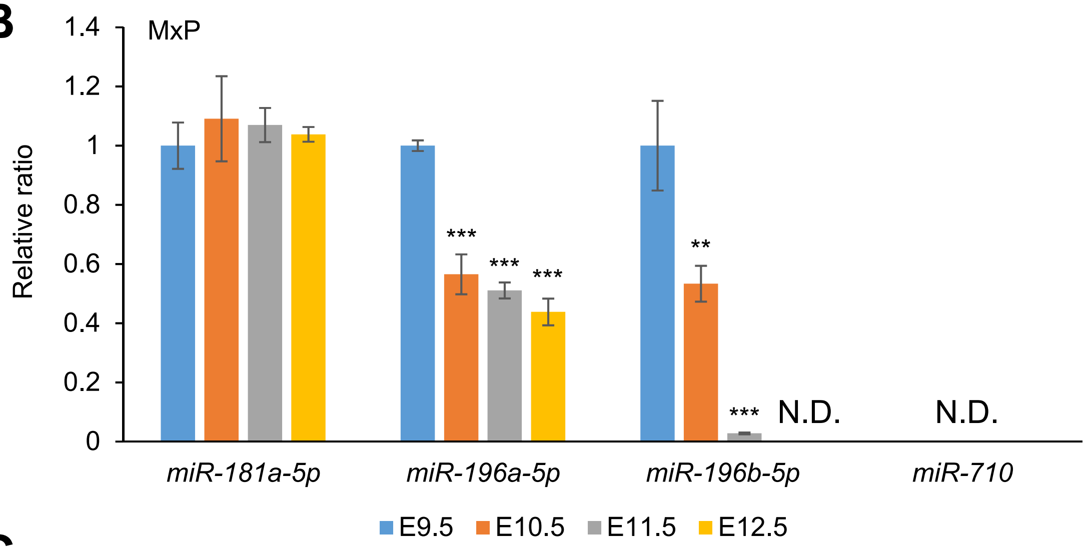

C

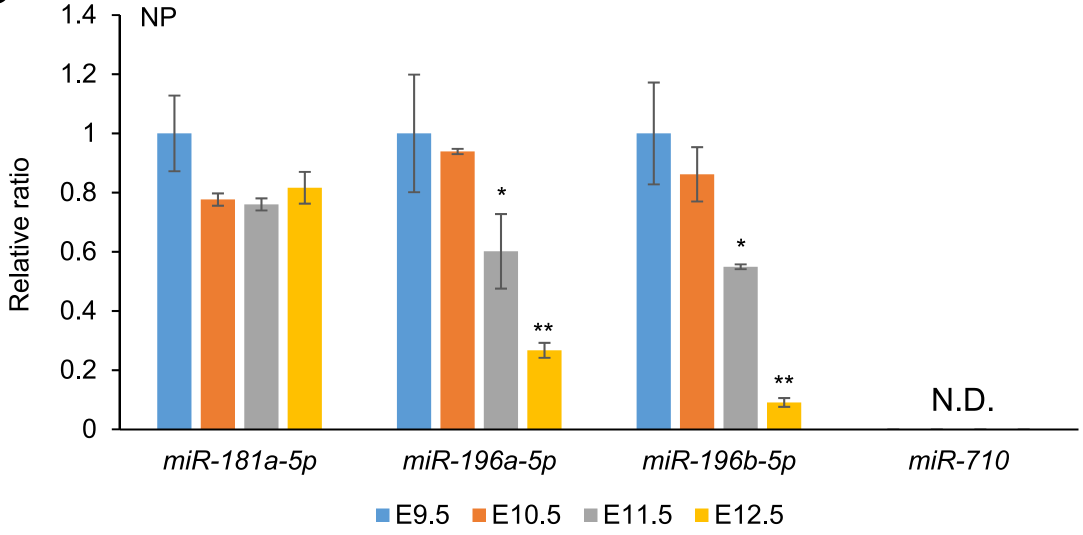

Supplementary Figure 4

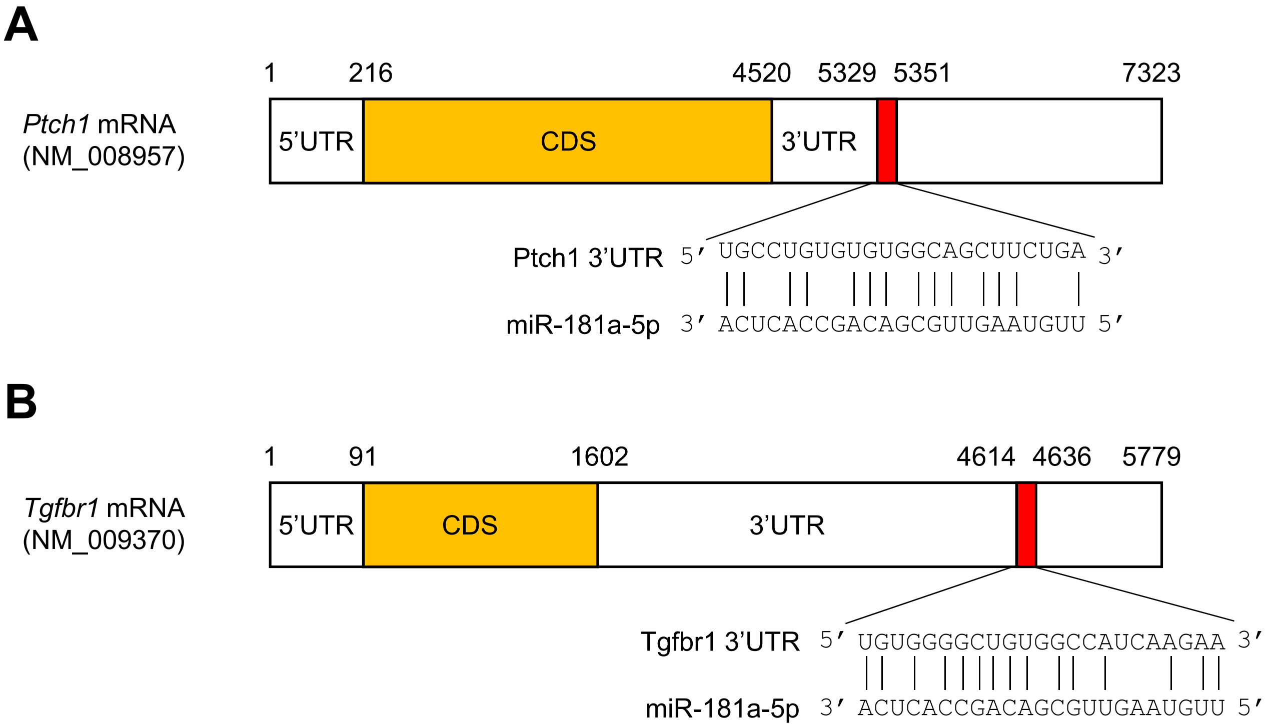

Supplementary Figure 5

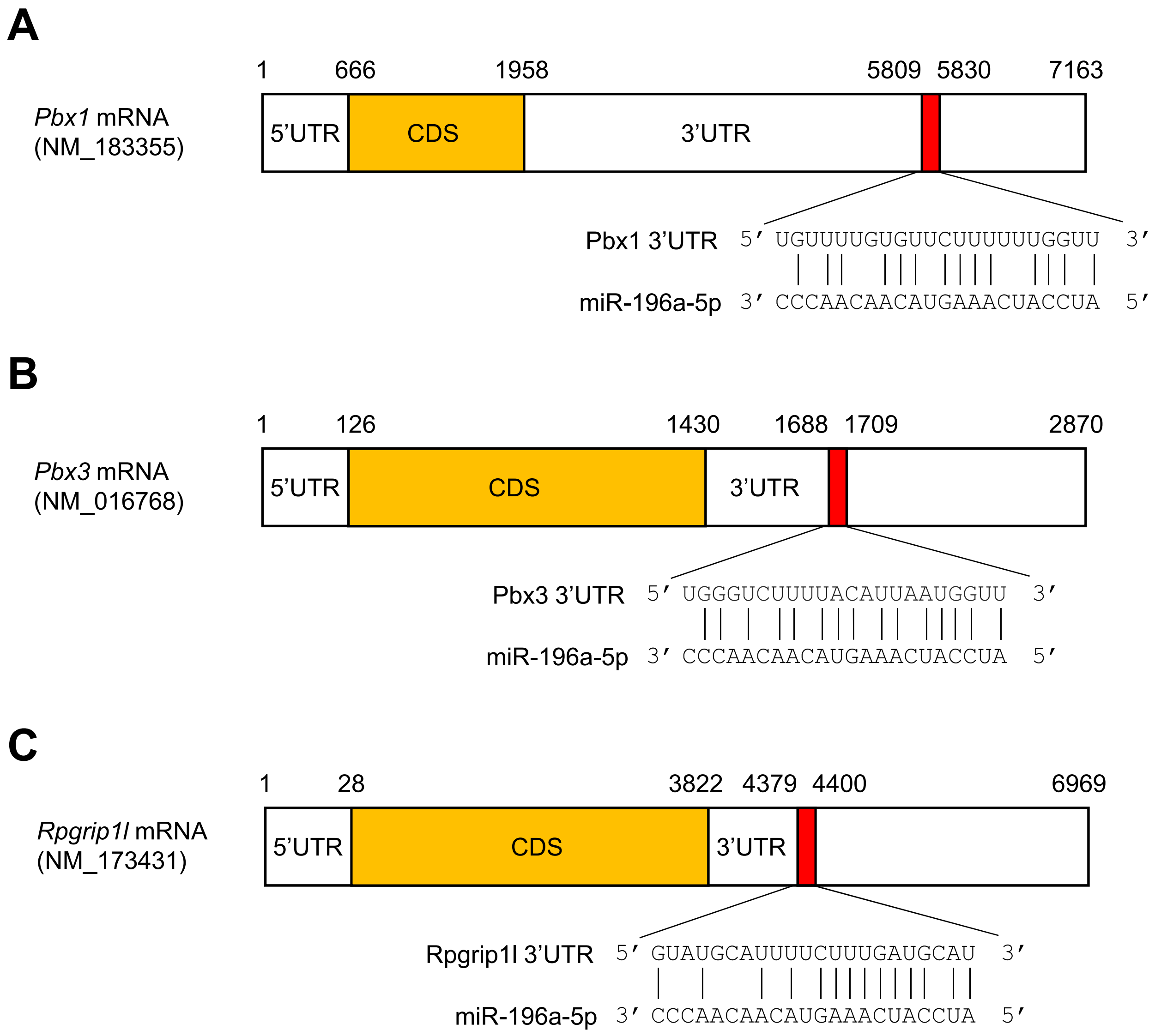

Supplementary Figure 6

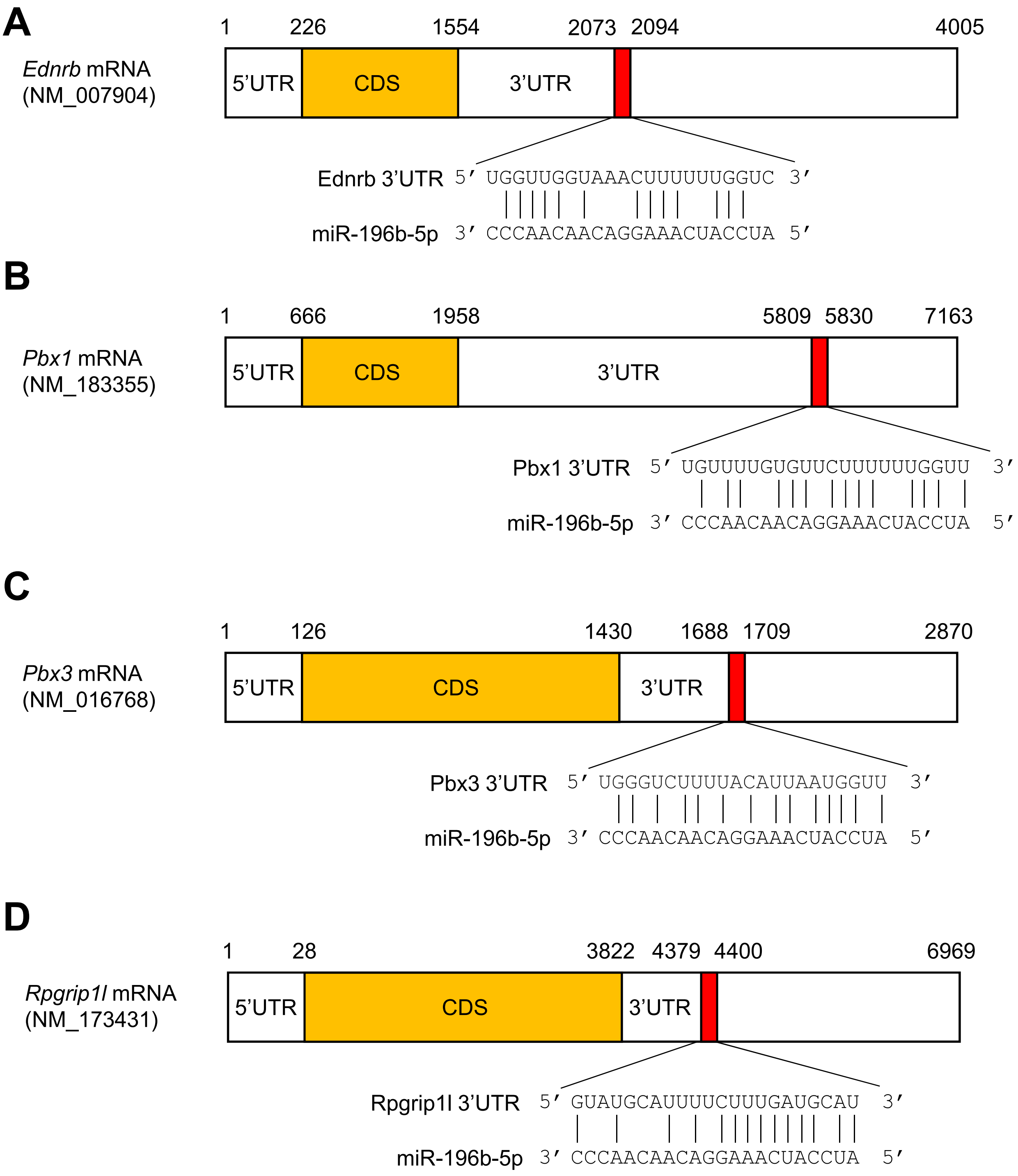

Supplementary Figure 7

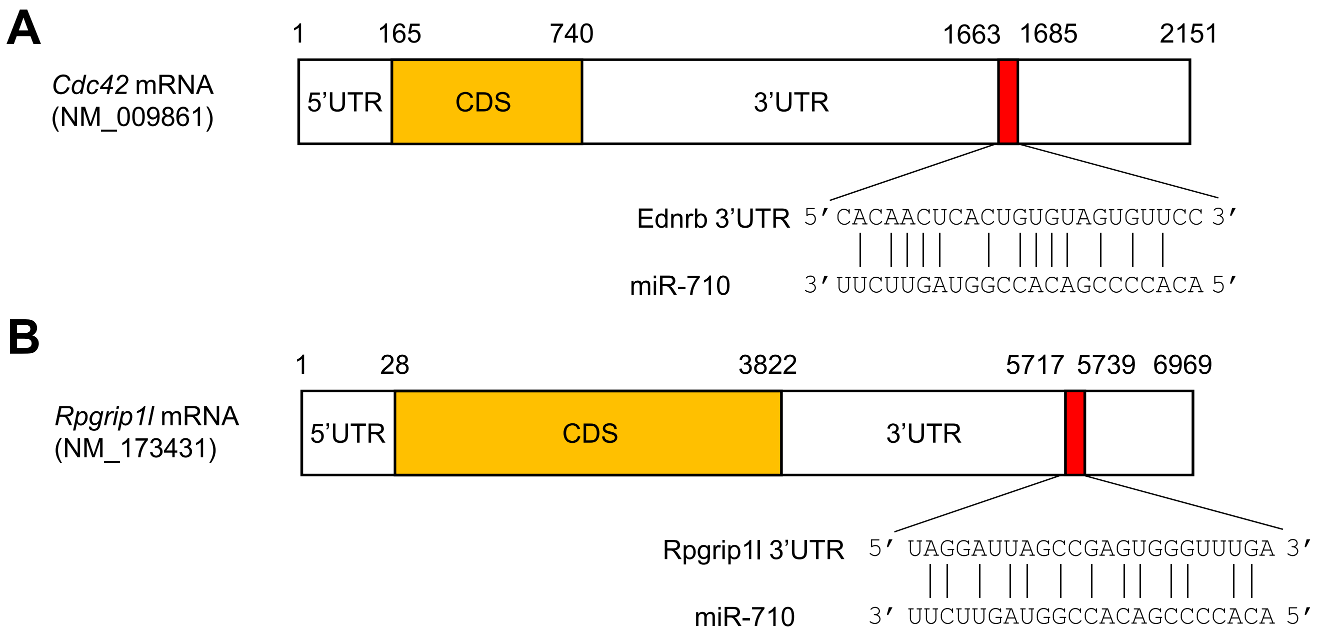

Supplement: Supplementary file 1 [file ijms-22-01746-s001.pdf]
